# Pathways to Effective Treatment for Individuals Convicted of Sexual Offences Who Are Taking Medication to Manage Problematic Sexual Arousal

**DOI:** 10.1177/10790632261460849

**Published:** 2026-06-17

**Authors:** Rebecca Lievesley, Kerensa Hocken, Belinda Winder, Christine Norman, Adarsh Kaul

**Affiliations:** 1 NTU Psychology, School of Social Sciences, 6122 Nottingham Trent University, Nottingham, UK; 2 Midlands Psychology Services, 57317 His Majesty’s Prison and Probation Service, Nottingham, UK; 3 Nottinghamshire Healthcare Trust, Nottingham, UK

**Keywords:** medication to manage problematic sexual arousal, sex offending, treatment, hypersexuality, sexual preoccupation, treatment pathways

## Abstract

The use of medication to manage problematic sexual arousal among individuals convicted of sexual offences is still an understudied treatment option in the U.K. Because of this, our knowledge of the medication service is limited to nomothetic analyses of pre- and post-treatment effectiveness, and qualitative analyses of the lived experiences of people taking the medication. This paper contributes a novel perspective on the service by using service-level data to chart the treatment pathways of all service users (*N* = 139) who have received this medication at the first evaluation site. Using a rigorous process of data triangulation and inter-rater reliability checking, we identified six distinct treatment pathways based on initial drug classes, changes in dosage or drug class, and the (dis)continuation of medication. Each pathway is associated with positive changes in indices of problematic sexual arousal, while highlighting the nuance within service user journeys. In conducting this exercise, we have also produced a framework for ethical client centered prescribing that considers both clinical effectiveness and service user experiences to help clinicians make decisions that are likely to yield both positive clinical outcomes and long-term medication adherence among service users.

When working with people who have sexually offended, a key factor to consider in treatment is problematic sexual arousal. Our use of ‘problematic sexual arousal’ reflects psychological states of excessive sexual thinking (i.e., sexual preoccupation) and engaging in excessive sexual behaviors to a degree that cause distress and discomfort to the individual concerned (i.e., sexual compulsivity or hypersexuality). In this context, ‘excessive’ does not have a specific clinical cut-off (as might be expected in a diagnostic context), but instead reflects a subjective experience whereby sexual arousal has a marked impact on everyday functioning (for a full discussion of this conceptualization, see Lievesley, 2019).

In this paper, we present an analysis of all cases of people with sexual convictions who are engaged in a pilot of a pharmacological treatment pathway for problematic sexual arousal. Our aim is to understand engagement with this pathway and to map service user journeys through the service^1^. The treatment pathway has not been examined in this way before, and as such this work represents a significant contribution to our understanding of this treatment pathway and the experiences of those referred to it. In presenting this analysis, we provide recommendations about the prescribing of such medication to people with sexual convictions.

## Contextualizing the England and Wales Protocol for the Pharmacological Treatment of Problematic Sexual Arousal

Despite being identified as key risk factors for both first time sexual offending (Finkelhor, 1984; Seto, 2019; Ward & Beech, 2017) and sexual, violent and general recidivism among individuals convicted of sexual offenses (Hanson et al., 2007; Hanson & Harris, 2000; Hanson & Morton-Bourgon, 2004; Knight & Thornton, 2007), most psychological treatment programs in England and Wales do not explicitly target hypersexuality or sexual preoccupation (see e.g., Elliott & Hambly, 2023)^2^. This potentially results in treatment needs relating to these issues being left unmet. This treatment ‘gap’ led to the suggestion that pharmacological treatment could provide a useful supplement to psychological programs. Consequently, protocols were established within the U.K. in 2007 to allow the voluntary use of pharmacological treatment of people with sexual convictions within the care of His Majesty’s Prison and Probation Service (HMPPS; Home Office, 2007). This is referred to as the medication to manage problematic sexual arousal (MMPSA) treatment pathway, and started being offered at HMP Whatton in 2009. The service has since been rolled out nationally as a treatment option in HMPPS and is now available in 12 prison establishments. The criteria for referral for the medication, as outlined by HMPPS (2008), includes evidence of one or more of the following:

• hyper-arousal (e.g., frequent sexual rumination, sexual preoccupation, difficulties in controlling sexual arousal, high levels of sexual behavior)
• intrusive sexual fantasies or urges
• sexual urges that are difficult to control
• sexual sadism or other dangerous paraphilias, or repetitive paraphilic offending such as voyeurism or exhibitionism.

The inclusion of paraphilic themes in the referral criteria maps on to many international prescribing protocols for the use of pharmacological treatment with people who have committed sexual offenses, and may be reflective of the prison-based context of the service, where offending behaviors are often intertwined with experiences of problematic sexual arousal. That is, many protocols aim to “replace paraphilic interests with sexual fantasies and behaviors that do not cause distress or impairment” (Winder et al., 2019, p. 162), and/or reduce the risk of sexual offending among those receiving such medication (e.g., Thibaut et al., 2010, 2020). This introduces a clear risk-based approach to prescribing, with the use of medications that can exert a progressively extreme effect on the ability to experience sexual arousal in response to an assess offending risk (see Bradford, 2001; Hill et al., 2003; Thibaut et al., 2010, 2020).

In contrast, the medication service within the U.K. is not risk-focused, and the prescribing protocol is instead based on a combination of the clinical indications and experiential effects of problematic sexual arousal, as defined at the outset of this paper, rather than on the treatment of paraphilias in isolation^3^ (Grubin, 2017, 2018). That is, it is not purely the presence of offense-related sexual interests that reflects eligibility for medication, but the experience of such interests as an issue affecting everyday functioning. This approach is designed to be a collaborative process between the prescribing professional and the service user, balancing the clinical needs of the case with the broader wellbeing of the individual (Grubin, 2018). For this reason, medication is recommended to be combined with psychological therapies (Grubin, 2017, 2018).

The MMPSA treatment pathway involves three main classes of medication: selective serotonin reuptake inhibitors (SSRIs; most commonly Fluoxetine), testosterone-lowering agents including anti-androgens (most commonly Cyproterone Acetate; CPA), and gonadotropin releasing hormone (GnRH) agonists. SSRIs are licensed antidepressant medications. Side effects include weight gain, headaches, fatigue, and sleep disruption (for a review, see Niarchou et al., 2024). Additionally, those taking SSRIs have reported issues related to reduced sexual arousal, sexual function, and ejaculation difficulties (e.g., Grubin, 2018; Niarchou et al., 2024). While SSRIs are not licensed for the management of sexual arousal (Jannini et al., 2022), there is an evidence base for their use in relation to hypersexuality (National Institute for Health and Care Excellence, 2015). Their use is also well documented in reducing offense related sexual interests and behaviors in patients with various paraphilias (Culos et al., 2024; Kafka & Hennen, 2000; Turner & Briken, 2021), the intensity of sexual fantasies and obsessions (Adi et al., 2002; Culos et al., 2024), general levels of sex drive (Landgren, Savard, et al., 2022b), and hypersexual behavior and sexual preoccupation among people with sexual convictions (Garcia & Thibaut, 2011; Winder et al., 2024).

Anti-androgens and GnRH agonists are found to moderate sex drive (Thibaut et al., 2010) and, as such, have a sound evidence base for treating hypersexuality and the intensity of paraphilic thinking and behavior (Briken & Kafka, 2007; Culos et al., 2024; Garcia & Thibaut, 2011; Jordan et al., 2011; Khan et al., 2015; Landgren et al., 2022a, 2022b; Turner & Briken, 2021), reducing symptoms associated with sexual preoccupation and hypersexuality (Bradford & Pawlak, 1993; Landgren et al., 2022a; Thibaut et al., 2010, 2020; Winder et al., 2024), and reducing both dynamic risk scores (Wolba et al., 2025) and rates of recidivism (Gallo et al., 2019; Thibaut et al., 2020) among people with sexual convictions. Given the higher potency of anti-androgens, regular medical monitoring is recommended during their use owing to the nature of the potential side effects. These include gynaecomastia (breast development), weight gain, reduced bone mineral density (osteoporosis), reduced testicle volume, and suicidality (for a full list of possible side effects and the requirements for medical monitoring, see Lippi & van Staden, 2017; Thibaut et al., 2020). These effects are considered to be similar to those of surgical castration, but are usually reversible within 4–8 weeks of discontinuation (Garcia & Thibaut, 2011; Grubin, 2018).

## The Need to Identify Treatment Pathways

Existing research into the use of medication with people who have sexually offended focuses on quantitative analyses of its effectiveness in relation to a range of clinical measures, either by comparing those taking different medication classes (Winder et al., 2014) or by examining differences between medicated and unmedicated individuals (Winder et al., 2024). Although this research is useful in eliciting findings regarding treatment effectiveness at the symptom level (e.g., Khan et al., 2015), it does not tell us about the unique and nuanced treatment pathways of individuals taking this medication. However, qualitative analyses of the lived experiences of people with sexual convictions taking selective serotonin reuptake inhibitors (SSRIs) and anti-androgens to treat their levels of sexual arousal (Lievesley et al., 2014, 2024) suggest that a ‘one size fits all’ approach is inappropriate (see also Thomas & Daffern, 2014). As such, a nuanced analysis of the journeys through the medication service (as is presented in this paper) provides a significant development in our understanding of the various changes that happen throughout treatment pathways.

According to Grubin’s (2017) guidance for the MMPSA treatment pathway, SSRIs are suggested as the most appropriate first line of treatment for those whose problems are primarily associated with sexual preoccupation, rumination, impulsivity, or mood dysregulation. Progression onto anti-androgens is recommended if there is an insufficient treatment effect. In contrast, those service users who report having difficulty in controlling their sexual urges, or who demonstrate high levels of sexual behavior (i.e., hypersexuality) are recommended to be treated with anti-androgens in the first instance, with progression to GnRH agonists in the case of an insufficient treatment effect. Despite the prescribing guidelines regarding the use of pharmacological treatments for this purpose, the individualized pathways of taking these medications are likely complex, but lack empirical exploration. For example, those referred to the medication service are known to change medication class or dosage, withdraw from treatment, return to treatment, and, for some, the medication does not reduce sexual arousal as desired (Elliott et al., 2018; Lievesley et al., 2013, 2014; Winder et al., 2014).

These different pathways are thus not reflected in the analyses of large nomothetic data sets. Although there are clear benefits to larger-scale investigations, looking at the detail of individual cases provides a different opportunity - to bridge the gap between scientist and clinician, and thus provide meaningful findings to inform practice (Hershenberg et al., 2012). This is particularly important in situations where treatment changes (in this case, to dosage or medication class) can be frequent or happen in a relatively short timeframe. When this happens, quantitative analyses using intention to treat criteria as a grouping strategy (e.g., Winder et al., 2014), and where data points are reported to be spaced at systematically separated time intervals (e.g., three, six, nine, twelve months, etc), risks missing the nuanced experiences and divergent journeys of service users.

## Aims of This Paper

This paper identifies the various pathways that are present within the MMPSA evaluation database and provides an individual case example to illustrate each of these. We aimed to capture the different pathways through the medication service to gain a clearer understanding of the effects, individuality, and complexity of the treatment service. This paper is thus less concerned with the specific effectiveness of medication in reducing measures of problematic sexual arousal (for these data, see e.g., Winder et al., 2014, 2024).

This paper contributes to a systematic analysis of the various journeys taken through the medication service. This analysis serves as a basis for understanding the complexity of the treatment offered to highlight (where necessary) issues with the heuristic initial prescription of medication in response to observable symptom profiles. That is, evidence suggests that some people start on too low (or too high) a treatment dosage or change drug class multiple times throughout their medication journey (Lievesley et al., 2014, 2024). In the analyses that follow we identify how these changes happen, and the effects of the medication among those within different treatment pathways.

## Method

### Sample Information

The sample for this study represents all participants in the national MMPSA evaluation database who had consented to receive medication (*N* = 139). Consent for inclusion in the evaluation dataset is assessed at the point of referral and in every meeting with the prescribing psychiatrist, who also ensures that service users’ engagement with the medication service is consistent with the voluntary nature of the pathway. The sample were all male, convicted and incarcerated for a sexual offense (or an offense with a sexual element), and had an average age of 48.10 years (*SD* = 13.76). Breaking the sample down by notable criminogenic characteristics where information was available, 6% were classified as low risk according to the RM2000 sex scale, 27% as medium risk, 39% as high risk, and 28% as very high risk. Most of the sample had offenses against children (72%), with smaller numbers offending against adults (24%) or both age groups (4%). Slightly more than half of the sample had offended only against female victims (54%), with 28% offending only against male victims and 18% offending against victims of both sexes. Exactly 50% of the sample were known to their victim(s), with 43% offending against strangers (7% of the sample offended against both known and stranger victims). Where prior treatment information was known, 90% of the sample had participated in some form of psychological intervention for people with sexual convictions (typically the former Sex Offender Treatment Programme run by HMPPS), and 40% had engaged with the Healthy Sex Programme for men with sexual interests that are directly linked to their offending.

### Data Sources

#### File Information

Information related to a participant’s referral for medication, as well as their treatment, monitoring, risk levels, and relevant life history (e.g. information relevant to the presence of problematic sexual arousal, or psychiatric diagnoses) was collated from file information and reports. These are routinely collected data, and are used to supplement the evaluation data (rather than being collected in the course of the evaluation itself).

#### Clinical Measures

Two brief scales (proposed by Grubin, 2008, 2017) were used to explore elements of problematic sexual arousal. Although these have not been empirically validated within the peer-reviewed literature, they form the basis of assessments for MMPSA services in the U.K. (see e.g., Scottish Forensic Network, 2022), and represent the clinical measures used to indicate changes in problematic sexual arousal among men engaging with the MMPSA treatment pathway. Hypersexual behavior was assessed with the following two items:

• Number of days in the past week masturbated to orgasm? (0–7 days)
• Number of days in the past week masturbated not to orgasm? (0–7 days)

Sexual preoccupation was assessed with the following three items:

• How much time do you spend thinking about sex? (0: Very little; 7: All the time).
• What is the strength of your sexual urges and fantasies? (0: Low; 7: High)
• What is your ability to distract yourself from sexual thoughts? (0: Easy; 7: Difficult).

### Procedure

Approval to conduct the research was initially granted by the Governor of the medication evaluation prison site. An application to conduct the research was made via HMPPS and the Nottingham Trent University Colleage of Business, Law and Social Sciences Research Ethics Committee. Once ethical clearance was granted, access to the participant data was granted by the prison establishment.

The data used is information that is routinely collected in the course of the MMPSA service evaluation. Specifically, individuals who consent to a referral for an assessment for medication (and subsequently consent to taking this) complete several clinical measures at regular intervals (see Lievesley et al., 2013). In addition, a range of pre-collected file information was collated for each case. Consent to access and use this data for research purposes is obtained at the point of referral for the medication as part of the evaluation of the service.

#### Development and Reliability of the Pathways and Case Studies

The process of identifying the pathways involved reviewing the medication journey for each individual that had received medication. Using the aforementioned data sources, particular features of each participant’s treatment experience were examined to identify specific treatment pathways. These features were:

(1) Changes and maintenance of medication class;
(2) Changes and maintenance of medication dosage;
(3) Medication starts, ends, and restarts.

Once the pathways were identified, a summary description (see Table 1) was developed for each. To enhance the rigor of our analysis an inter-rater reliability assessment was undertaken. This took the form of the first author and one independent researcher reviewing every case (*N* = 139) against the summary descriptions of each pathway and allocating to one of the pathways. Twelve cases were excluded prior to the analysis due to incomplete or inconsistent information. These cases should not be viewed as ‘attrition’ in a traditional sense, as their removal from the analysis stems from a lack of data being collected during the prescription of medication. There is nothing about these cases (e.g., age, risk level) to distinguish them from those with more complete data. As such, 127 cases were included in the analysis and were allocated to a pathway independently by both raters. Inter-rater reliability was strong (Cohen’s κ = .87), with 91.4% agreement in pathway classifications. In the 11 cases (8.6%) where raters disagreed, pathway assignment was subsequently decided through a discussion after jointly examining the clinical data.

**Table 1.** Description of Representative Medication Treatment Pathways

Pathway	Description
1: SSRI pathway	The service user begins SSRI medication. There may be medication adjustments (i.e. to the specific type of SSRI prescribed or dosages), however they remain on SSRIs for the duration of their treatment. Successful completion of this pathway would involve ceasing the medication when problematic sexual arousal is at a desirable and manageable level. Any deviation from this would result in movement to another pathway.
2: Anti-androgen pathway	The service user begins anti-androgen medication. There may be medication adjustments (i.e. to the specific type of anti-androgen prescribed or dosages), however they remain on anti-androgens for the duration of their treatment. Successful completion of this pathway would involve ceasing the medication when problematic sexual arousal is at a desirable and manageable level. Any deviation from this would result in movement to another pathway.
3: Progression pathway	The service user begins medication – either SSRIs or anti-androgens. There may be medication adjustments within this medication class (i.e. to the specific type prescribed or dosages), before progressing to a stronger medication class (i.e. anti-androgens or GnRH agonists) potentially alongside the initial prescribed medication. Once on this, there may be further medication adjustments within this medication class. Successful completion of this pathway would involve remaining on this stronger medication class until the point of ceasing the medication when problematic sexual arousal is at a desirable and manageable level. Any deviation from this would result in movement to another pathway.
4: Switching pathway	The service user begins medication – either SSRIs or anti-androgens. There will be multiple adjustments to the prescribed medication class (i.e. between SSRIs, anti-androgens and GnRH agonists, or a combination of these in either direction) within their treatment journey. There may also be medication adjustments within each medication class (i.e. to the specific type prescribed or dosages). Successful completion of this pathway would involve remaining on medication (any class of medication) until the point of ceasing the medication when problematic sexual arousal is at a desirable and manageable level. Ceasing the medication prematurely would result in movement to another pathway.
5: Drop-out pathway	The service user prematurely (i.e. before desired effects on problematic sexual arousal have been achieved) ceases medication due to a lack of desired effect, or the presence of unwanted effects (i.e. side effects), while on one of the above pathways.
6: Re-entry pathway	The service user re-starts some form of medication after ceasing treatment in any of the above pathways.

The reliability of pathway classification was also checked using detailed interrogations of case information related to each case within the dataset. Structured discussions also took place with the prescribing psychiatrist regarding each case example to assess the accuracy of the information being presented and the interpretations being made. This process was performed to ensure the reliability of the discussion and recommendations presented later in this paper. As such, we believe that the pathways identified in our analysis are reliable and valid, and that our classification of cases to pathways has been conducted using a rigorous process.

Once the pathways were confirmed, one representative case study example for each pathway was selected from the full dataset of participants to illustrate each of the pathways. Although each representative case is not comprehensive in covering every possible circumstance of each pathway, the essence of each pathway is present in these examples. These specific cases were chosen due to having the most complete, relevant and up to date data to develop a case study that was relevant to each pathway. To protect confidentiality, identifying details have been omitted. These case studies are provided in the supplemental materials.

## Results

**Figure 1.**
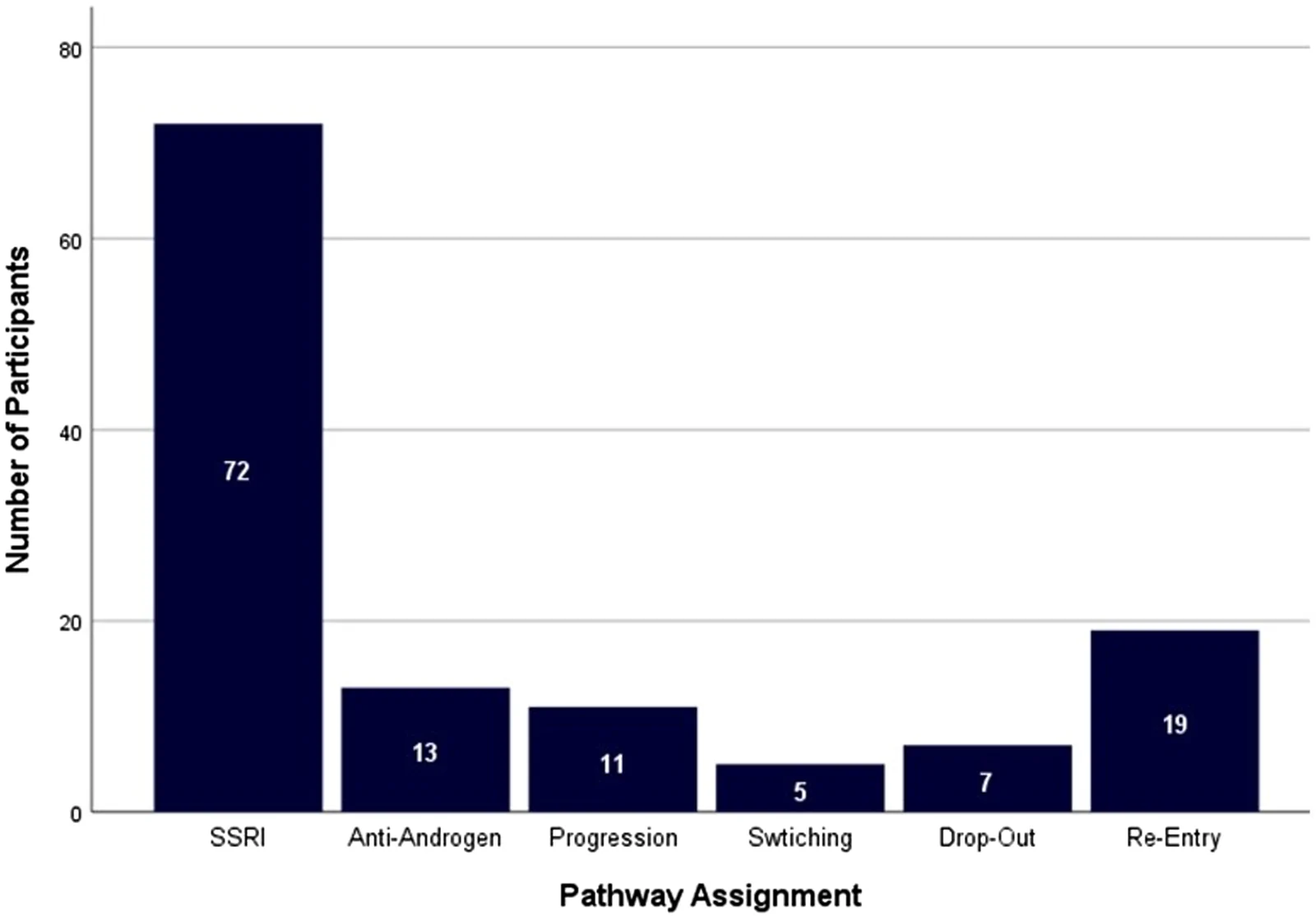
Distribution of pathway assignment

Six core pathways were identified as being representative of those having received medication to manage their sexual arousal. The summary descriptions of these are presented in Table 1. Figure 1 represents the distribution of cases classified into each pathway.

### Discussion of the Pathways

#### Pathway 1: SSRI Pathway

Pathway 1 *(the ‘SSRI pathway’)* involves only the use of SSRIs as a form of medication. Individuals on this pathway will begin on SSRIs, and while the dosage may be adjusted as necessary to achieve the desired effects, they will remain on SSRIs until medication is no longer required.

Individuals initially placed on this pathway reported high levels of the clinical measures of sexual preoccupation prior to commencing medication (pre-medication *M* = 4.82, *SD* = 1.28). A reduction in these measures was observed during treatment, with reports of reduced sexual thoughts and fantasies, and greater control over those that they do experience (most recent measures; *M* = 1.40, *SD* = 1.25, *t* (69) = 17.18, *p* < .001, *d*_z_ = 2.05). Clinical measures of hypersexual behavior (operationalized masturbation to orgasm; Kafka, 1997; Långström & Hanson, 2006),) are also addressed by SSRI treatment for those on this pathway (pre-medication *M* = 4.03, *SD* = 2.35; most recent measure *M* = 1.05, *SD* = 1.38; *t* (68) = 11.80, *p* < .001, *d*_z_ = 1.42). This contradicts the basic assumptions within the literature and prescribing guidelines, which argue that hypersexuality should be treated using anti-androgen medication in the first instance (Grubin, 2017). Effect sizes of the differences between the beginning of treatment and the most recent measures are very large.

Individuals on the SSRI pathway also report improvements in mood and general psychological wellbeing in a manner consistent with the more traditional use of SSRIs for emotion regulation (Cools et al., 2008; Skandali et al., 2018). This reduction in indicators of problematic sexual arousal, as well as improvements in general wellbeing, provides individuals on this pathway with the headspace necessary to actively engage in rehabilitation through psychological treatment programs, when previously problematic sexual arousal would have presented as a barrier to this by interfering with engagement (Lievesley et al., 2014; Saleh et al., 2010). This is particularly important for individuals on this pathway with SSRIs affecting levels of neural plasticity, thus increasing sensitivity to the environment and making individuals more susceptible to psychological therapy and more amenable to change (Branchi, 2011; Carhart-Harris & Nutt, 2017).

#### Pathway 2: Anti-androgen Pathway

Pathway 2 *(the ‘anti-androgen pathway’)* involves only the use of anti-androgens as a medication class. Individuals on this pathway will begin on anti-androgens, and while the dosage may be adjusted if necessary in order to achieve the desired effects, they will remain on anti-androgens until medication is no longer required.

Individuals on this pathway demonstrated high levels of all clinical measures pre-treatment, and observed very large reductions in these during the treatment period (sexual preoccupation: pre-medication *M* = 5.57, *SD* = 1.00; most recent measure *M* = 1.88, *SD* = 1.15, *t* (11) = 9.24, *p* < .001; *d*_z_ = 2.67; hypersexuality: pre-medication *M* = 5.05, *SD* = 2.02; most recent measure *M* = 2.10, *SD* = 2.28, *t* (9) = 3.82, *p* = .004; *d*_z_ = 1.21). However, anti-androgen treatment is associated with potentially severe side effects (Nguyen et al., 2015), with individuals on this pathway reporting problematic effects including an inability to gain or maintain an erection or reach orgasm, and gynecomastia (breast growth). These complications are evident in the case example (see supplemental materials). Such effects may be a cause for concern for individuals taking it with this leading to lower levels of compliance, or engaging in more deviant or risky sexual fantasizing in order to achieve sexual satisfaction for some individuals experiencing these effects (Lievesley et al., 2014). As such, more regular or intensive monitoring of the effects and thus compliance may be necessary for individuals on this pathway.

#### Pathway 3: Progression Pathway

Pathway 3 *(the ‘progression pathway’)* involves the progression from one medication class to a stronger medication class (i.e. from SSRIs to anti-androgens, from anti-androgens to GnRH agonists, or a combination of two medication classes) due to a lack of desired effect on levels of problematic sexual arousal. While there may be additional dosage adjustments or further progression, individuals on this pathway will remain on a stronger medication class than they were initially prescribed until the medication is no longer needed.

Individuals on this pathway either observed no effect from their initially prescribed medication, or observed some effect but felt that this was insufficient (clinically or subjectively by the service user). This often leads to a dosage increase of the initial medication, followed by a progression to an alternative medication class if sufficient improvements are not observed (potentially alongside the initial medication class i.e. combing SSRIs and anti-androgens). Within this pathway, there may be a clinical decision for individuals to pause the medication to allow safe and successful movement onto another medication class. However, the intention to continue taking medication remains, meaning that this does not constitute treatment drop-out.

Although there are too few participants currently on this pathway to conduct meaningful statistical analysis, the trends in relation to clinical measures of sexual preoccupation (pre-medication *M* = 5.55, *SD* = 0.92; most recent measure *M* = 2.24, *SD* = 2.19) and hypersexual behavior (pre-medication *M* = 4.65, *SD* = 2.65; most recent measure *M* = 0.90, *SD* = 1.60) appear promising.

#### Pathway 4: Switching Pathway

Pathway 4 *(the ‘switching pathway’)* involves the commencement of medication using either SSRIs or anti-androgens. Due to a lack of desired effect on an individual’s experience of problematic sexual arousal, or the experience of side effects, adjustments to dosage or medication class (both up and down in terms of strength) are required. Individuals on this pathway experience numerous changes to the medication class and dosage until the medication is no longer needed.

For individuals on this pathway, the ‘switching’ is often linked initially to a lack of (sufficient) improvement. This may lead to a dosage increase or a progression from, for example, SSRIs to anti-androgens (the progression pathway). Similarly, for individuals on anti-androgens but still observing problems in relation to measures of sexual preoccupation, SSRIs may then be employed to attempt to address these concerns. However, alongside this is the need to balance the adverse effects of the medication, for example, the introduction of a stronger class of medication may lead to unwanted side effects (e.g., gynecomastia, or total loss of sexual function associated with anti-androgens; Lievesley et al., 2024; Nguyen et al., 2015) resulting in further dosage or medication class changes in an attempt to address these. Working with the prescribing health professional in order to balance both the targeted and undesired effects is vital in order to find a suitable combination. As with the progression pathway, there may be a clinical decision for individuals on this pathway to pause the medication to allow safe and successful movement onto another medication class. However, the intention to continue taking medication means that this brief cessation of treatment does not constitute drop-out.

Although there are too few participants currently on this pathway to conduct meaningful statistical analysis, the trends in relation to clinical measures of sexual preoccupation (pre-medication *M* = 5.75, *SD* = 0.55; most recent measure *M* = 1.69, *SD* = 1.84) and hypersexual behavior (pre-medication *M* = 3.75, *SD* = 3.78; most recent measure *M* = 0.50, *SD* = 0.58) appear promising.

#### Pathway 5: Drop-Out Pathway

Pathway 5 *(the ‘drop-out pathway’)* involves the commencement of medication, with adjustments to dosage or medication class as necessary to attempt to achieve the desired reduction in problematic sexual arousal or overcome unwanted side effects but this is not achieved before the individual prematurely ceases taking the medication (i.e., before desired clinical effects have been afforded adequate time to be achieved).

The most common reason for this treatment drop-out is related to the undesirable effects of the medication, such as gynecomastia or inability to achieve or maintain an erection. Regarding drop-out that is related to a lack of desired effect on indicators of problematic sexual arousal, it is acknowledged that there is no biological explanation as to why medication should not have some effect on these clinical outcomes. This is particularly the case for those receiving anti-androgens or GnRH agonists, both of which directly affect levels of testosterone, and thus a sufficient dose would eliminate physical arousal (Thibaut et al., 2010, 2020; Winder et al., 2019) and should therefore impact upon the clinical measures related to hypersexuality. Individuals on this drop-out pathway may not fully engage with this process, and instead choose to discontinue the medication without allowing sufficient time for improvements to be observed. Thus, premature discontinuation may be related to motivation or readiness to change (Lievesley et al., 2014).

While there are too few participants currently on this pathway to conduct meaningful statistical analysis, the clinical measures of sexual preoccupation (pre-medication *M* = 4.34, *SD* = 1.67; most recent measure *M* = 3.97, *SD* = 1.07) and hypersexual behavior (pre-medication *M* = 4.06, *SD* = 3.27; most recent measure *M* = 3.33, *SD* = 2.94) do show some reduction over time, although to a much smaller degree than the other pathways.

#### Pathway 6: Re-Entry Pathway

Pathway 6 *(the ‘re-entry pathway’)* involves the commencement, ceasing and subsequent re-starting of medication. Individuals on this pathway may cease the use of medication either because they reach a level of arousal that they feel is now manageable without medication (which may be due to the medication or engagement with psychological treatment), or due to the reasons discussed within the drop-out pathway (Pathway 5). However, medication is then re-started due to the recognition that it was a premature discontinuation and that medication is still required.

Individuals on this pathway will often have observed improvements in the clinical measures related to their sexual thoughts and behaviors while taking medication at an earlier point of their treatment journey, leading to the belief that their sexual arousal is no longer problematic, and leading them to cease the medication. However, for some individuals, this discontinuation occurs too soon (i.e., before they are able to appropriately manage their sexual arousal independently).

The data in relation to clinical measures of sexual preoccupation (pre-medication *M* = 4.98, *SD* = 1.33; most recent measure *M* = 1.48, *SD* = 1.46; *t* (18) = 8.55, *p* < .001; *d*_z_ = 1.96) and hypersexual behavior (pre-medication *M* = 3.87, *SD* = 2.69; most recent measure *M* = 0.63, *SD* = 0.96; *t* (18) = 5.64, *p* < .001; *d*_z_ = 1.29) show that positive effects can be achieved after resuming medication.

## Discussion

This paper has addressed a current gap in the literature surrounding the use of medication with people who have sexually offended by examining journeys through this emergent treatment service. In doing so, we have identified the different treatment pathways within the U.K. service. This process was undertaken using rigorous processes to gain a clearer understanding of the individuality and complexity of treatment pathways that are missed within large-scale nomothetic evaluations of medication effectiveness with this population.

Six pathways were identified using data from the full sample of those receiving medication, related to SSRI and anti-androgen use, progression, switching between medications, drop-out, and re-entry. These have been discussed above and illustrated using a representative case example for each (see supplemental materials). This discussion will now consider the implications of the identified pathways on practice before discussing the relevant limitations.

### Reflections on the Current Prescribing Guidelines

According to prescribing guidelines for the use of medication within the U.K. treatment service (Grubin, 2017), direct entry onto anti-androgens is recommended for individuals who demonstrate an exceptionally strong sexual drive, high levels of sexual activity (hypersexual behavior), or risk-related behaviors that are difficult to control (Grubin, 2017; Winder et al., 2019). However, there are no specific criteria or clear thresholds within the guidance for what constitutes an ‘exceptionally strong’ sexual drive or ‘high’ levels of sexual activity. As such, this process of deciding who meets these criteria relies on the clinical judgment of the prescribing health professional, leading to subjectivity in the decision as to whether SSRIs or anti-androgens are the most appropriate first-line option for medication. In some circumstances this may be considered a strength as it allows clinicians to take individual service user preferences and subjective experiences of problematic sexual arousal into account (see Lievesley et al., 2014, 2024) when deciding between the medication classes. However, this may also be problematic and lead to individuals being prescribed anti-androgens with potentially severe side effects (Nguyen et al., 2015; see also Lievesley et al. (2024) for narrative accounts of these adverse and unwanted effects) when SSRIs could have the same clinical benefits in addressing all aspects of their problematic sexual arousal (as indicated within the above identified pathways). Furthermore, the existing guidelines do not consider the prior or current use of SSRIs for co-morbid conditions (e.g., depression), which is something that should be considered when making a decision regarding their suitability as a first line option. This is because a previous or current prescription for SSRIs that has not been accompanied by reductions in clinical indicators of problematic sexual arousal suggests that this class of medication may be insufficient for the individual under consideration, leading the service user to require a stronger medication class (i.e., anti-androgens).

As identified in the introduction to this paper, there is also inconsistency in relation to various prescribing protocols that are used internationally. For example, SSRIs are used as a first line of treatment within the U.K.’s MMPSA service for those whose primary clinical indication is sexual preoccupation (Grubin, 2017), as well as for individuals with low level paraphilic interests who are not deemed to be at high risk of reoffending within the WFSBP protocol (Thibaut et al., 2010, 2020). Despite this, others suggest that SSRIs should not be used as a primary treatment option for this purpose as it can be addressed more effectively with anti-androgens and GnRH agonists (Winder et al., 2019). Further, there are concerns related to both the aims of treatment and prescribing practices when considering the use of medication with people with sexual convictions. According to Winder et al. (2019) an aim of pharmacological treatments in this context is to replace paraphilic interests with alternatives that are not associated with distress, personal impairment, or increased risk of sexual offending. Other protocols have the aim of totally eliminating the capacity for sexual arousal in those who pose the highest risk of sexual offending (Thibaut et al., 2010, 2020).

From a client centered perspective, there is an argument to be made that the least severe class of pharmacological treatment should be used in order to achieve the required clinical effect. For example, SSRIs are known to address problematic sexual arousal in a manner that still allows for some degree of sexual life (Lievesley et al., 2014), which is particularly important when sexual satisfaction is cited as a primary human good associated with greater levels of long-term service user wellbeing and reductions in re-offending likelihood (Ward & Marshall, 2004). This consideration of the strengths of the various medication classes (i.e., their effects on physiological sexual arousal) and the subsequent implications that this has on everyday functioning and personal identity is consistent with a health-based approach to prescribing. That is, contrary to the risk-related aims of the prescribing protocols described above, the goal should ultimately be to prescribe the least potent class of medication that is needed to help service users to achieve their personal treatment goals, rather than to over-prescribe a stronger class and titrate this as treatment progresses. With this in mind, there is a case to be made that, unless otherwise indicated (e.g., due to a prior history of prescription), SSRIs are uniformly the most suitable first-line form of medication.

### Implications of the Current Findings: A Framework for the Use of Medication

**Figure 2.**
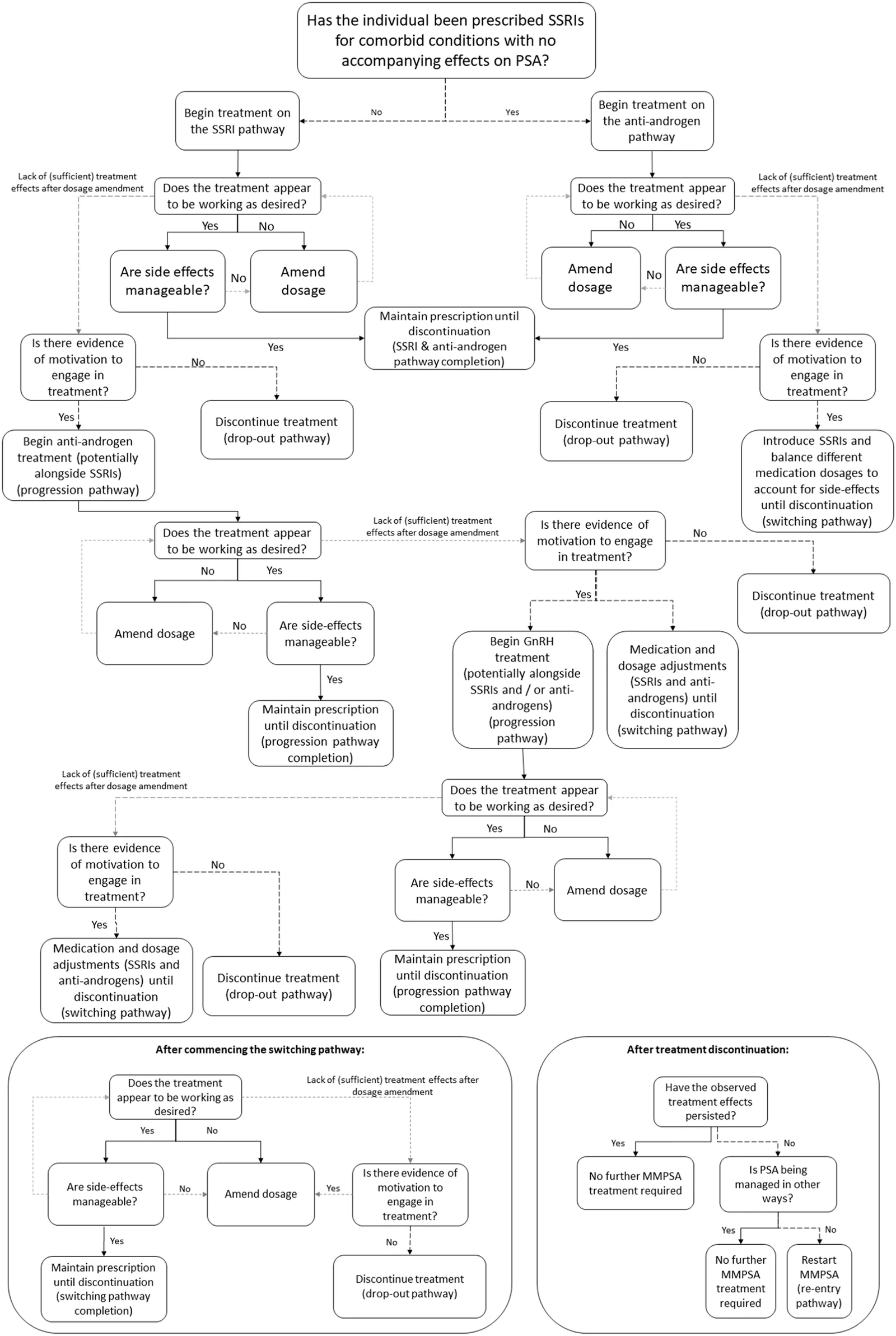
Recommended pathways through medication treatment (solid black arrows = within-pathway decisions; dashed grey arrows = within-pathway assessments; dashed black arrows = between-pathways movement)

It is clear that there is a need to standardize a coherent and ethical approach to prescribing medication to people with sexual convictions, and the pathways presented here provide the first comprehensive evidence base to be able to begin to achieve this. Through an analysis of the pathways, and consideration of the possible movement between them, we have developed a framework of prescribing that represents the first comprehensive guide to aid clinicians’ decision making in this context. A flowchart describing this framework is presented in Figure 2.

Based on the concerns outlined above regarding the current prescribing guidelines and protocols, coupled with the findings presented here, it is recommended that, where possible, all individuals begin their medication treatment journey on the SSRI pathway. There will naturally be exceptions to this, for example, where SSRIs have previously been (or are currently being) prescribed for co-morbid conditions (e.g., depression) without observing any clinical changes in problematic sexual arousal, or where clinical judgment suggests this would not be appropriate. In such circumstances it is recommended that individuals commence their treatment journey on the anti-androgen pathway (unless there are contraindications to this, such as pre-existing health conditions [e.g., high liver enzymes, severe cardiac disease, or meningioma] that would be exacerbated by anti-androgen medication; for a full discussion of contraindications for all relevant medications, see Thibaut et al., 2020).

Analyses of the data here show that those on the SSRI pathway demonstrate a very large and statistically significant reduction on the clinical measures related to measures of hypersexuality and sexual preoccupation, with these differences being in line with the size of the effects observed among participants on the anti-androgen pathway. With the comparability of these indices of clinical effectiveness in mind, and acknowledging that service users who take SSRIs report less severe side effects and a great intention to comply with medication in the longer term (as compared to those taking anti-androgens; Lievesley et al., 2014, 2024), it is therefore a logical first step to use SSRIs in the first instance with people experiencing problematic sexual arousal. Where this medication is not sufficient, movement onto the progression pathway (i.e., the addition of anti-androgens) should then be considered. This also fits with service user preferences, with a recognition that if making an informed decision, service users would typically choose SSRIs over other treatment options (Bourget & Bradford, 2008; Winder et al., 2019). This change to ensure the least severe class of medication is used in the first instance provides a much more client centered approach and moves the treatment of problematic sexual arousal away from a risk-based approach, and more congruent with a health-based approach to prescribing, which is essential when considering the presenting problem is primarily medical. Changes back to SSRIs should then be considered when supplementary psychological therapies have been engaged with, and sexual arousal has become easier for the service user to manage with less severe medication. At the heart of this process is a collaborative approach to prescribing, and any medication switching should be undertaken following discussions between the service user and prescribing psychiatrist, and always with the consent of the service user. This helps to ensure that service users are an active partner in the prescribing process, which is known to improve intrinsic motivations to continue pharmacological treatment when external pressures (e.g., a prison sentence, in the current context) are removed (for recommendations as to how this may be managed in relation to the use of medication with people who have sexually offended, see Lievesley, 2019).

These findings also call for the need for thorough monitoring of individuals throughout their medication treatment. This would allow easy monitoring of the effects of SSRIs, and thus if the SSRI pathway is not observed to be effective, changes in dosage or medication class (and thus movement through the pathways as discussed above) can be easily actioned until the desired treatment effects are achieved, ensuring that individuals are not confined to particular pathways. The importance of careful monitoring is also paramount for those ceasing medication to ensure that this is undertaken in a controlled way, monitoring any signs of potential relapse (e.g., the return of symptoms of problematic sexual arousal) and the individuals’ ability to manage these without the medication. Within the current sample, all incidents of relapse were effectively managed and medication was appropriately re-started (Pathway 6) due to the ability of service users and professionals to recognize the return of symptoms, and the relatively straightforward availability of the medication. However, this may not be as straightforward in a less controlled and therapeutic environment (i.e. in the community), and it is not possible to know the extent to which the restrictive nature of prison impacted rates of sexual behavior in the current sample. Future work on the continuation of treatment into the community upon release should be considered a priority area of evaluation. This would help to understand the extent to which the prison context is (1) a determining factor in pathway progression, and (2) impacting experienced levels of problematic sexual arousal.

### Limitations and Recommendations for Further Pathway Validation

The research here forms part of the national evaluation of the U.K.’s MMPSA treatment pathway (Lievesley et al., 2013), and uses clinical measures that are routinely collected as part of the treatment service. As such there was little control over the collection of data to ensure that it was regular and consistent across the sample. In practice, this means that some participants did not have data available for all time points, with the time points being unique to each participant based on clinical need (as they were collected at each meeting with the prescribing psychiatrist). Added to this, from the current data it is not possible to identify exactly when any change in medication commenced. This is because the data collected regarding medication class and dosage at the time points indicate what the participant is on at that specific time, and so where a change in medication occurs between two time points we know that the prescription was amended at the first, and was started sometime before the second. This lack of chronological information makes it difficult to draw definitive conclusions about the speed at which the medication was seen to be (in)effective, and when dosages were actually adjusted (impacting the precision of the switching and progression pathways). The collection of additional data regarding when prescriptions were collected, and when the new medication commenced would overcome this limitation.

These inconsistencies in data collection via the national evaluation also made it difficult to conduct formal quantitative analyses of participants’ journeys, as single case design analysis using either inferential statistics or formal visual inspection methods (Kazdin, 2011) require systematic, consistent data collection, and clearly defined phases of treatment (Nock et al., 2007). As such, this study relied on visual inspections of graphical data whereby trends were observed at a global level and interpreted in a descriptive way. This was also an appropriate approach to use in this study as specific ratings of clinical effectiveness were not the outcome being sought. Instead, this study was designed to explore the various journeys and experiences of individuals taking medication in relation to their starting points, navigation through various medications and dosages, and their eventual exit routes away from the treatment. Despite the subjectivity of this analytic approach, the span of the identified pathways feels comprehensive, which provides confidence in the validity of our conclusions. Nonetheless, improving data collection procedures over the longer term to ensure a more consistent approach to measuring change over time will improve the inferences that can be made about service user progress across each of the identified pathways.

Finally, it is important to acknowledge the relatively small sample size for identifying distinct pathways through a treatment option. This is due to an assignment to one of the latter four pathways (progression, switching, drop-out, or re-entry) being contingent on a service user’s experiences of either the SSRI or the anti-androgen pathway in the first instance, and their total time taking medication. As such, with the passage of time it seems inevitable that numbers on these latter pathways will increase. While the six pathways described here do seem to have face validity as the only possible journeys through taking medication (when considering logical medication changes and discontinuation routes), it is possible that other pathways were not represented in this particular dataset, and may be uncovered with a larger sample as the medication service becomes more established as a treatment option for people with sexual convictions who are exhibiting problematic sexual arousal. As such, as larger samples do become available through the national roll-out of the medication service it would be advisable to test these pathways against formal quantitative cluster analytic techniques to confirm them as the true structure of the service.

Each of these limitations point to the need for large-scale and properly-designed randomized controlled trials of medication to manage problematic sexual arousal. Such trials would allow for a more structured approach to evaluating the effectiveness of an MMPSA treatment pathway, including the standardization of data collection intervals. An experimental approach such as this would provide an opportunity to disentangle the effects of psychological interventions and engagement with pharmacological treatment. It is also important to control for the effect of engaging with interventions specifically designed to recondition paraphilic or offense-related sexual interests. That is, interventions involving directed masturbation (e.g., the Healthy Sex Programme) may inadvertently indicate an increase in problematic sexual arousal (through the appearance of higher masturbatory activities), despite this reflecting positive engagement with targeted treatment approaches.

Studies looking at the effectiveness of the medication treatment should also incorporate more sensitive measures of problematic sexual arousal. At present, the clinical measures used in the MMPSA evaluation lead to ceiling effects, where the maximum score of seven equates to the number of days whereon a particular service user masturbated. As such, for some participants this would equate to seven orgasm events (or total sexual outlets) per week if masturbating once per day. However, for others, this frequency may be much higher (e.g., our ‘Mr. E’ case example on the drop-out pathway, who reported masturbating up to 15 times per day; see supplemental materials). In this context, ‘number of days’ is not a sensitive enough indicator of sexual activity to detect significant changes in sexual behavior (e.g., ‘Mr. E’ could cut his masturbatory activity to five times per day – a drop of up to 70 total sexual outlets per week when using his upper estimate of daily orgasms – but remain scoring at the level of ‘7’ on the evaluation’s clinical measure). More sensitive measures (e.g., involving diary-keeping of actual numbers of masturbatory activities) might be combined with more psychological measures of the quality, experience, and everyday impact of sexual arousal to provide a more complete picture of the effects of medication in this context.

### Closing Statement

This paper has addressed a current gap in our understanding of the use of medication with people who have sexually offended by examining individuals’ journeys, and identifying pathways of treatment within the U.K. treatment service. In doing so, we have been able to make a significant contribution to this area of both research and practice in identifying how clinicians can work in ethical and clinically effective ways, and in a manner that is collaborative with service users.

## Supplemental Material

Supplemental Material - Pathways to Effective Treatment for Individuals Convicted of Sexual Offences Who Are Taking Medication to Manage Problematic Sexual Arousal Supplemental Material for Pathways to Effective Treatment for Individuals Convicted of Sexual Offences Who Are Taking Medication to Manage Problematic Sexual Arousal by Rebecca Lievesley, Kerensa Hocken, Belinda Winder, Christine Norman, and Adarsh Kaul in Sexual Abuse

## Notes

1. It is worth noting at the outset that our analyses are derived from routinely collected data from the MMPSA treatment service.

2. Although Elliott and Hambly’s (2023) report on the Horizon and Kaizen treatment programmes cite ‘healthy sexual interests’ as a treatment target, this is operationalized as reducing the use of sex as a coping strategy. As standard, these programmes focus on healthy sexual and social relationships and addressing offense-supportive cognition, rather than looking to reduce sex drive or preoccupation in a direct way.

3. Treatment of offence-related sexual interests as a specific need is undertaken using the Health Sex Programme (for a summary, see Hanley, 2025).
