# Supplementary material for: Pathways to Effective Treatment for Individuals Convicted of Sexual Offences Who Are Taking Medication to Manage Problematic Sexual Arousal
Source: Sex Abuse. 2026 Jun 17;38(7):798–821. doi: 10.1177/10790632261460849 (PMC13534773; doi:10.1177/10790632261460849)
Supplement: Supplementary file 1 [file sj-pdf-1-sax-10.1177_10790632261460849.pdf]

Pathways to effective treatment for individuals convicted of sexual offences who are taking medication to manage problematic sexual arousal – Supplementary material

Pathway 1: SSRI Pathway.

Case example: Mr A

History of PSA

Mr A reports having prolonged and unmanageable sexually violent fantasies, and frequently using sex or masturbation as a method of coping with negative emotions and managing these sexual thoughts. Mr A reports being in numerous relationships with repeated unfaithfulness, often engaging in several overlapping sexual relationships.

MMPSA treatment journey

Fluoxetine 20mg → Fluoxetine 40mg

Mr A was initially prescribed a daily dose of SSRIs (Fluoxetine) 20mg. After approximately three months this was increased to 40mg as, while there had been a reduction in measures, these were not considered to be sufficient by Mr A or the psychiatrist and some measures had begun to increase again (T0[1]-T3). Within the first 6 months of being on this increased dosage, measures of PSA (both sexual preoccupation and hypersexuality) reduced to zero or one (between T3-T9) and stabilised at this low level. At T18 there was a slight increase in number of days masturbated not to orgasm from zero to one however, as this was still low, this was not considered to be problematic. Mr A remained on 40mg of Fluoxetine and had been taking this for almost two years when he left the evaluation site (moved to a category D establishment) but planned to remain on this.

[1] Throughout, 'T' is used to refer to the time interval whereby T0 is baseline (prior to beginning any medication) and all other number refer to the length of time since commencing medication (i.e. T1=1month on MMPSA, T3=3months on MMPSA etc).

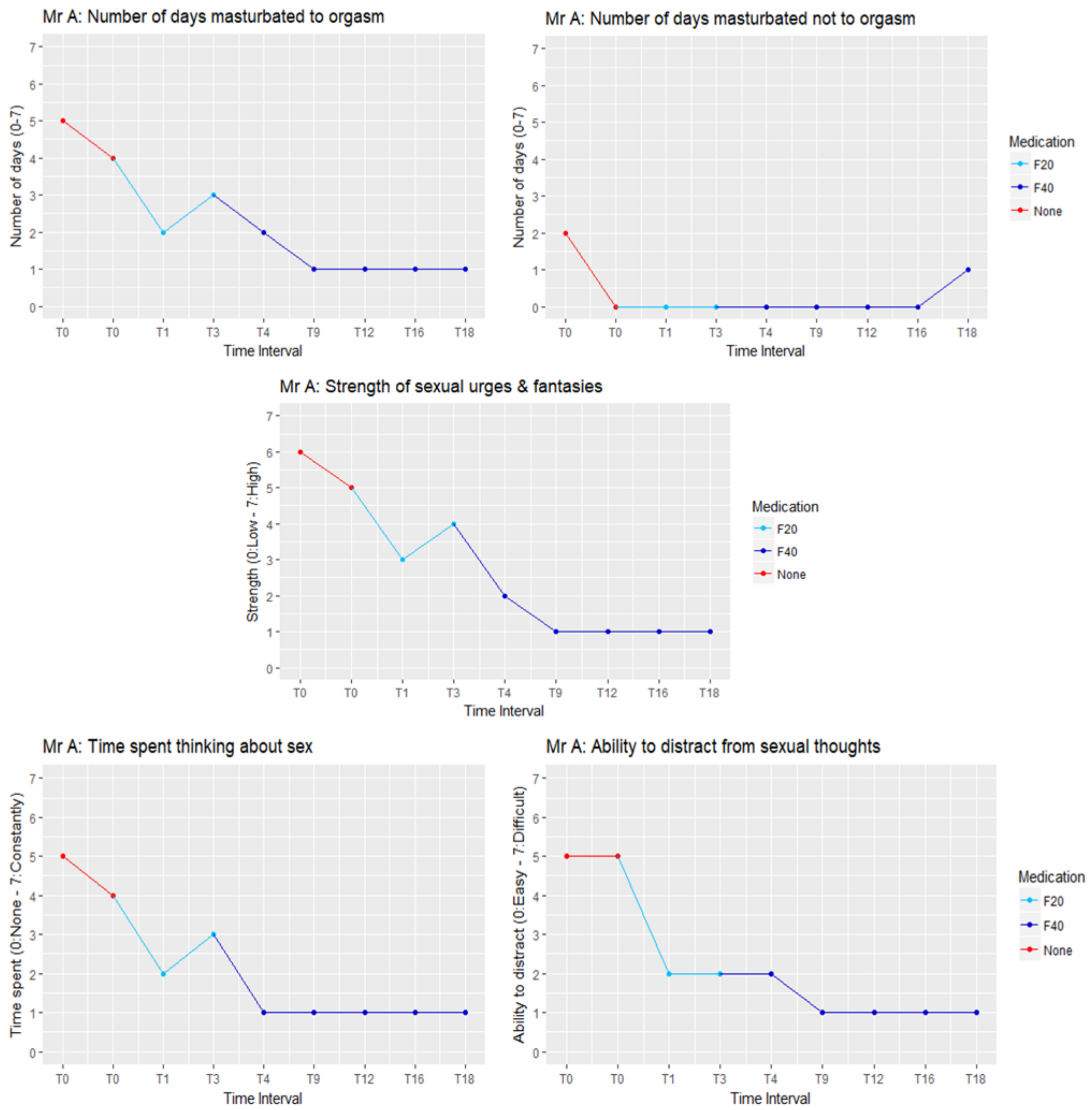

Figure 1. Changes in Mr A's clinical indicators of PSA

Mr A's case demonstrates an example of SSRIs (Fluoxetine) having the desired effect of reducing levels of PSA (both hypersexual behaviour and sexual preoccupation). Stabilised scores of one across a range of measures indicate that Mr A still has some sexual thoughts and is able to masturbate to orgasm, but that these are manageable (i.e. they are low in strength and easy to distract from). This is particularly positive as the aim of the medication is to allow individuals to maintain a healthy level of appropriate sexual functioning (Bradford, 2001; Thibaut et al., 2010). Furthermore, accounts of individuals receiving SSRIs for this purpose indicate that concerns around their ability to have a sexual relationship in the future may result in non-compliance or ceasing the medication (Lievesley et al., 2013; 2014). It is also worth noting that according to the current prescribing guidelines (Grubin, 2017), Mr A should have commenced treatment on anti-androgens based on his presenting symptoms of PSA (high levels of hypersexuality, offence related fantasies and risk related behaviours), however, due to medical reasons he instead commenced on SSRIs which have been successful in managing his level of PSA.

Pathway 2: Anti-androgen pathway

Case example: Mr B

History of PSA

Mr B began engaging in sexual activity from a young age (before 10 years of age), masturbating several times per day and regularly using pornography. Mr B reports having over 30 sexual partners, with only two significant relationships. While in custody, Mr B reports having unmanageable sexual thoughts, being easily sexually aroused and masturbating multiple times per day.

MMPSA treatment journey

Androcur 50mg → Androcur 100mg → Androcur 50mg→ Stopped

Mr B was initially prescribed a daily dose of anti-androgens (Androcur) 50mg. This was due to very high levels of clinical measures of both hypersexual behaviour and sexual preoccupation which is in line with prescribing protocols for MMPSA (e.g. Grubin, 2017). In addition, he had previously taken SSRIs for the treatment of depression but had observed no change in his sexual thoughts and arousal. Despite some improvement on this medication, Mr B was still experiencing intrusive sexual thoughts and arousal. Levels of masturbation to orgasm remained high, and masturbation not to orgasm increased from a pre-medication level of zero to seven (T0-T4). After approximately four months, this was increased to a daily dose of 100mg which led to a decrease in all clinical measures from T4 onwards. Mr B remained on this for almost 2.5 years (until T33) and while measures of PSA fluctuated within this period, all measures remained below a score of two and were not considered problematic by the psychiatrist or Mr B. While the medication was considered to be working well in relation to clinical indicators, Mr B had begun to develop gynecomastia as a result of prolonged use of the anti-androgens (this is a documented side effect of this class of medication; Nguyen et al., 2015). As such, at T33 the prescribed dosage was reduced to 50mg. During this period, the scores fluctuated but remained low with Mr B reporting that he still felt he was able to manage his sexual thoughts and arousal. He remained on 50mg for a further 22 months before raising further concerns about the side effects, particularly gynecomastia. As Mr B had been on the medication for over 4.5 years, was experiencing concerning side effects and felt that he could cope without it, it was decided that he would stop all medication. Mr B has now been off the medication for seven months and has shown no increase in measures of PSA during this time, with some continuing to decrease (T55 onwards).

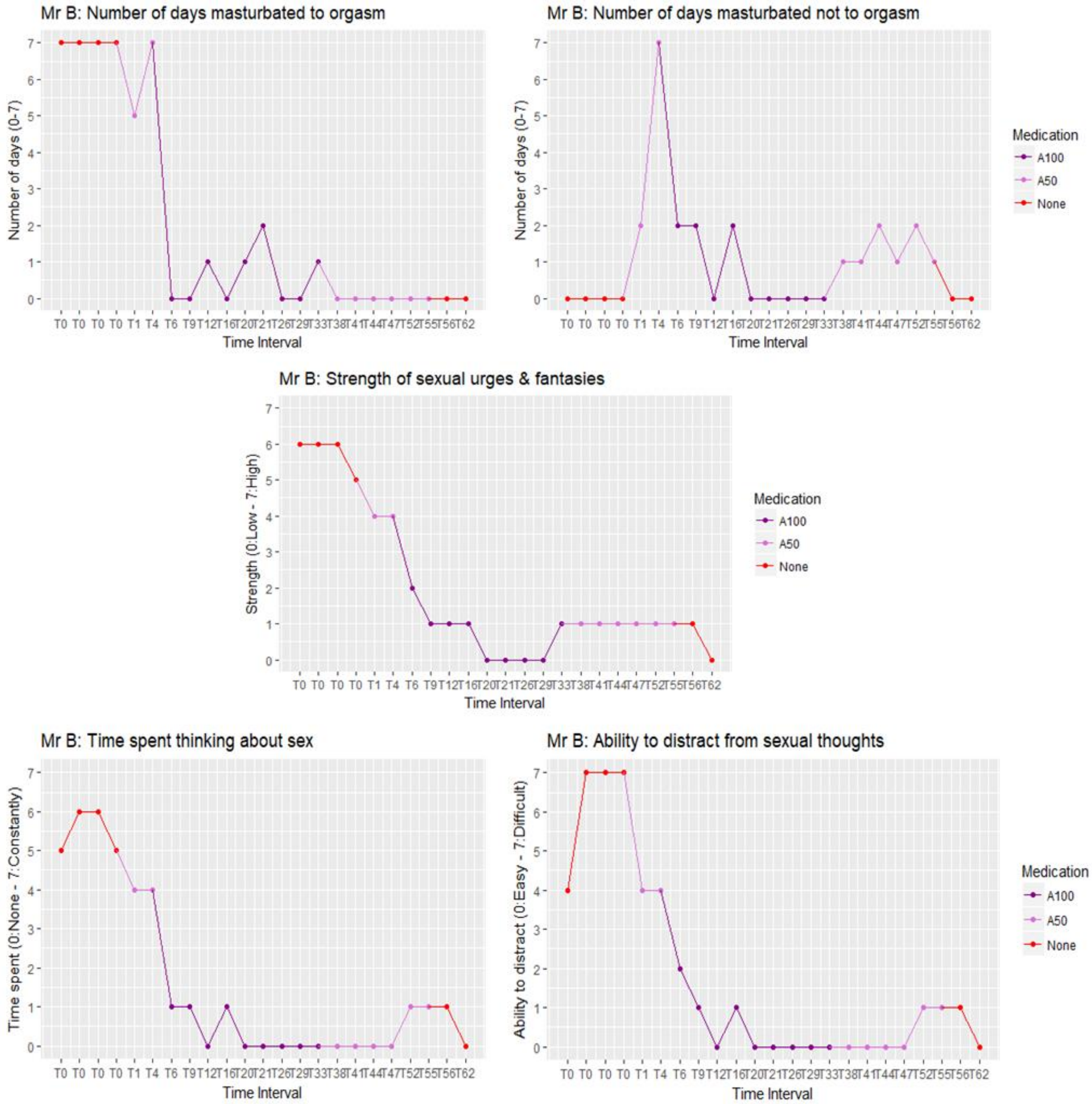

Figure 2. Changes in Mr B's clinical indicators of PSA

Mr B's case demonstrates an example of anti-androgens (Androcur) having the desired effect of reducing all levels of PSA (hypersexual behaviour and sexual preoccupation). This allowed Mr B to engage effectively in HSP and develop effective coping mechanisms and strategies to manage his sexual thoughts and arousal independently so he was able to successfully withdraw from the medication. Stabilised scores of one or zero across a range of measures indicate that Mr B still has some sexual thoughts but that these are manageable, being low in strength and easy to distract from. These are positive effects of the medication, in that they reduce the intensity of sexual thoughts while still allowing an acceptable amount to be experienced. However, self reported scores related to masturbation behaviours indicate that Mr B struggles to masturbate to orgasm after beginning the medication. This is demonstrated through the fluctuations in levels of masturbation not to orgasm, where masturbation is attempted, but orgasm is not achieved. This is concerning as the aim of the

medication is to reduce inappropriate or problematic sexual thoughts and behaviours while still allowing individuals to maintain a healthy level of sexual functioning (Bradford, 2001). Thus, removing the capacity for sexual outlets could impact on the individuals wellbeing, with sexual satisfaction being considered a primary human good (Ward & Marshall, 2004). While this may not have been problematic for Mr B thus far, and he is no longer on the medication, this could present concerns for the future. Such problems may lead to increased fantasies, sexual frustration, and non-compliance (e.g. stopping the medication in order to achieve orgasm or engage in future sexual relationships), as has been demonstrated in others receiving MMPSA (Lievesley et al., 2014, 2024).

Pathway 3: Progression pathway

Case example: Mr C

History of PSA

Mr C has a sexual preference for children between the ages of 4-13. He reports thinking about sex daily, finding his sexual fantasies overwhelming. This sexual preoccupation was present in the lead up to his sexual offending, with his drinking also exacerbating his sexual fantasies and leading to a lack of control. Mr C has reported that sex makes him happy, which is what led him to seek this at the time of his index offence.

Mr C reports being in one serious relationship with another male which lasted for four years, before breaking down due to his current conviction. He reports that both he and his partner were unfaithful, acknowledging that he regularly engaged in sexual activity with other men, and was also being paid for sex.

MMPSA treatment journey

Fluoxetine 20mg → Fluoxetine 40mg → Fluoxetine 20mg → Fluoxetine 40mg → Paused → Androcur 50mg → Androcur 100mg → Androcur 50mg

Mr C was initially prescribed a daily dose of SSRIs (Fluoxetine) 20mg, following which he experienced decreases in all clinical measures reported here, aside from ability to distract from sexual thoughts, which remained the same. Shortly after starting the medication, Mr C commenced HSP and reported struggling with the requirements of the course and increased focus on his sexual thoughts and masturbation and requested that the dosage be increased to 40mg for the duration of his engagement in HSP. This increased dosage led to further decreases in clinical measures, or a maintenance of the scores already achieved through his 20mg dose. The most notable change was with his ability to distract from sexual thoughts which decreased from a score of seven to a score of zero. Once HSP was completed, the dosage was returned to 20mg of Fluoxetine (T7), however, shortly after this reduction, measures of PSA (aside from masturbation not to orgasm) largely increased (T7-T10) to pre-medication levels. This led to a return to his 40mg dose and decreases were seen once again. Despite these reductions, the extreme fluctuations in scores between dosage changes, and inappropriate behaviours being observed and reported by staff, led to the decision that anti-androgens may be more appropriate to better manage his sexual thoughts and arousal. Mr C paused all medication for a period of time due to complications with other medications which resulted in an observed increase in all measures except masturbation not to orgasm (T13-T15), before beginning a daily dose of anti-androgens (Androcur) 50mg at T15. As such, his gap in medication was not a cessation of treatment, but rather a necessary pause in order to allow the safe and effective switch from SSRIs to anti-

androgens to take place, alongside his other medications. This led to a reduction in measures over the first four months, with all scores reaching zero, however by T31, after being on Androcur 50mg for approximately 16 months all scores had again increased, with Mr C reporting difficulty managing his sexual thoughts and arousal, resulting in a dosage increase to 100mg. Mr C remained on this for approximately ten months before requesting a dosage decrease due to concerns regarding side effects, particularly gynecomastia. The dosage was decreased to Androcur 50mg (T41) and he remained on this for approximately six months before moving to a category D establishment and while he intended to remain on this, it is unclear whether he did so.

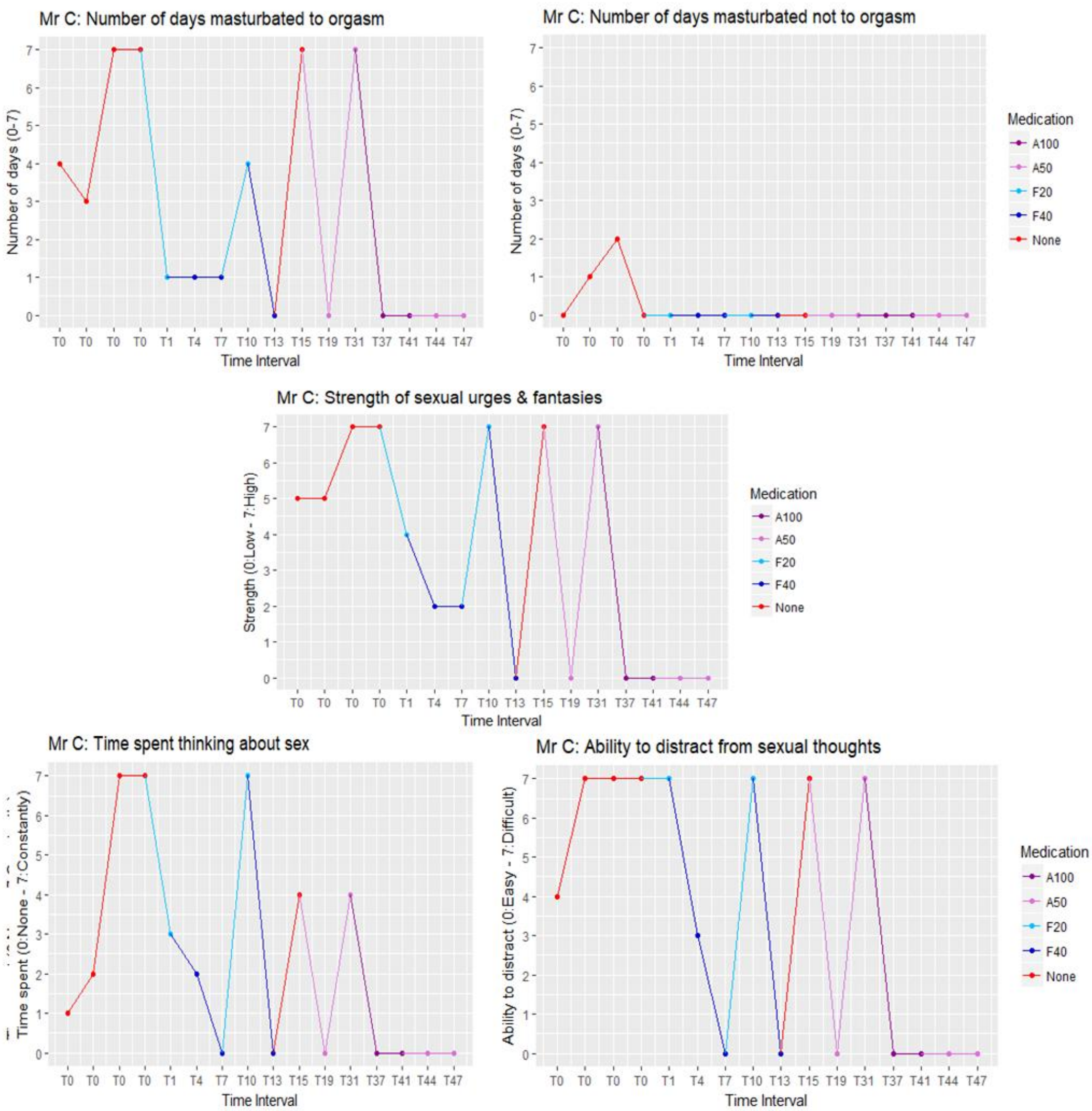

Figure 3. Changes in Mr C's clinical indicators of PSA

Mr C's case demonstrates an example of dosage adjustments and a progression from SSRIs to anti-androgens to sustain prolonged improvements in the management of PSA. This case clearly illuminates how these decisions and processes are not simply based on reported levels of problematic arousal, but instead need to take account of other demands and factors that could contribute to this, in this case for example, engagement with HSP. The combined engagement in HSP alongside

medication in this case was problematic, with the aims of HSP requiring individuals to focus on developing healthy sexual fantasies and incorporating the use of directed masturbation. Naturally, engagement in this process increased scores across the clinical measures and so the understanding the context of this is key to determining the extent to which this is problematic. In such cases, the aims of HSP and the medication could indeed be competing against each other and so this is something to must be carefully monitored and managed as in this case. Furthermore, it is also possible that the use of the medication may prevent active engagement in programmes such as HSP if it impacts upon the ability to gain and maintain an erection, which is a recognised side effect of anti-androgens (Nguyen et al., 2017). In addition, this case highlights how despite a suitable medication being found (Anti-androgen, Androcur, 100mg) to manage levels of arousal, the known side effect of such medications mean that this is not a long term viable option and result in further adjustments in order to manage the undesired effects of the medication.

Pathway 4: Switching pathway

Case example: Mr D

History of PSA

Mr D reports using pornography and masturbating daily from the age of twelve. His first sexual relationship was at the age of 17. He has had a total of seven sexual partners in his life, although five of these were casual or one night stands. He reports having two relationships but was unfaithful throughout both. Mr D reports having regular sexual thoughts about adults and children that he finds difficult to distract from, becoming easily aroused and masturbating several times per day while in the community and in prison.

MMPSA treatment journey

Fluoxetine 20mg → Fluoxetine 40mg → Fluoxetine 20mg & Androcur 50mg → Androcur 50mg → Fluoxetine 20mg & Androcur 50mg → Androcur 100mg → Androcur 50mg

Mr D was initially prescribed a daily dose of SSRIs (Fluoxetine) 20mg. While he experienced some decrease in reported levels of the more psychological aspects of PSA (T0-T1), this was considered to be insufficient and the dosage was subsequently increased to 40mg (SSRI; Fluoxetine) after approximately one month. Over the next two months (T1-T3), the reported levels of the clinical measures either remained stable or increased, resulting in the decision to progress to a combination of SSRIs (Fluoxetine, 20mg) and anti-androgrens (Androcur, 50mg). This led to a decrease in all clinical measures of PSA (except masturbation not to orgasm which remained at zero) over the next four months (T3-T7) however, by T11 a number of the measures had begun to increase. By this point, Mr D was reporting side effects of tiredness and headaches which were believed to be the result of the SSRIs, resulting in a discontinuation of these from T11, and thus a daily dose of anti-androgens (Androcur) 50mg. Over the next four months (T11-T15), while some measures decreased, his rates of masturbation and strength of sexual urges and fantasies continued to increase, resulting in the decision to return to a combination of SSRIs and anti-androgens at T15. This led to an initial reduction in clinical measures, which then fluctuated over the next eight months (T15-T23) coupled with continued side effects assumed to be associated with SSRIs, Mr D again requested to discontinue the SSRIs. At this point (T23) the dosage of anti-androgens was increased to 100mg due to concerns that 50mg alone had previously been insufficient. He remained on this for 11 months and while clinical scores fluctuated within this period, Mr D did not consider these changes to be problematic. This resulted in the dosage being reduced to 50mg (Androcur) at T34 due to the belief that Mr D was managing his arousal well. Mr D had been on this for approximately 20 months, with the clinical

measures decreasing and stabilising at a manageable level over this period, when he was released into the community with the intention to continue the medication.

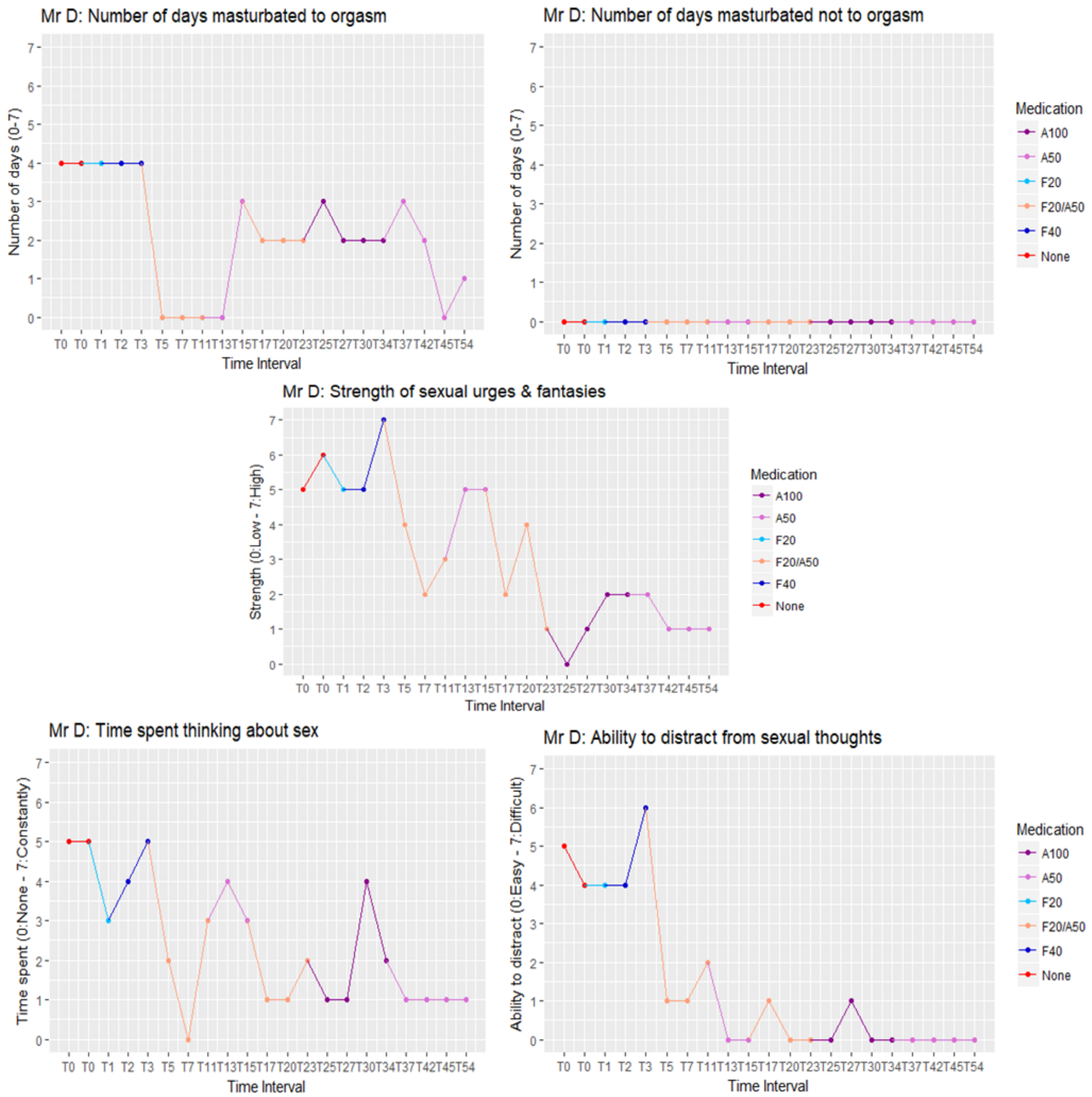

Figure 4. Changes in Mr D's clinical indicators of PSA

This case demonstrates an example of the need to make frequent adjustments to both medication class and dosage in order to balance the desired effects on PSA, alongside the undesired effects of the medication. Mr D embodies this switching approach with six 'switches' over a 54 month period since his initial prescription of SSRIs. As can be seen in Figure 5, indicators of his PSA were unaffected by MMPSA until anti-androgens were added to his prescription. However throughout his treatment journey was characterised by repeated switching between various combinations of medication class and dosages. This constant switching in the absence of substantial movements in indicators of PSA indicates the result of careful monitoring on the part of the professionals charged with managing

individuals like Mr E. While the effects of MMPSA on clinical indicators of PSA appear to be driven by the presence of anti-androgens, these are accompanied by negative side effects (Nguyen et al., 2015). In balancing these positive and negative effects in collaboration with the individual taking MMPSA, it is possible to work together in relation to acknowledging the achievements of the medication in bringing PSA under control. On stronger MMPSA (e.g. doses of CPA at 100mg) there will naturally be experiences of side effects that play a role in the motivations of individuals taking such medications to continue to engage in treatment (Lievesley et al., 2014, 2024). This is where we observe reductions in dose, or the attempted incorporation of SSRIs, into the prescription.

Pathway 5: Drop-out pathway

Case example: Mr E

History of PSA

Mr E reports engaging in sexual activity with his partner between one to five times per day. In addition, he would also masturbate up to 15 times per day and reports being easily aroused and having an interest in sexual violence. He reports regularly using pornography, particularly that of an extreme or violent nature (including materials depicting bestiality). While in custody, Mr E reports having constant offence related sexual thoughts and masturbates 10-15 times per day, often waking in the night for this purpose. Despite this, he reports that his sexual thoughts and behaviours are something that he enjoys.

Treatment journey

Androcur 50mg → Androcur 100mg → None

Mr E was initially prescribed a daily dose of Androcur 50mg. This was due to extreme levels masturbation and offence related sexual fantasies that he found difficult to manage. After approximately one month, there had been no marked improvement, with the measures of PSA remaining the same (T0-T1). After his first month on medication, due to a lack of desired effect Mr E's dosage was increased to 100mg of Androcur, which he remained on for a further four months. However, he reported no substantial changes in any of the clinical measures during this period, with all of these remaining high (save for a small decrease in the amount of time spent thinking about sex (T1-T3). Throughout the period of receiving medication, Mr E reported numerous side effects that he considered to be unmanageable including weight gain and headaches. As a result of this, and believing that the medication was having no impact on his levels of PSA (as observed in relation to the clinical measures), he subsequently decided to discontinue the medication after five months (the graphs only illustrate three months as Mr E did not attend further follow up meetings).

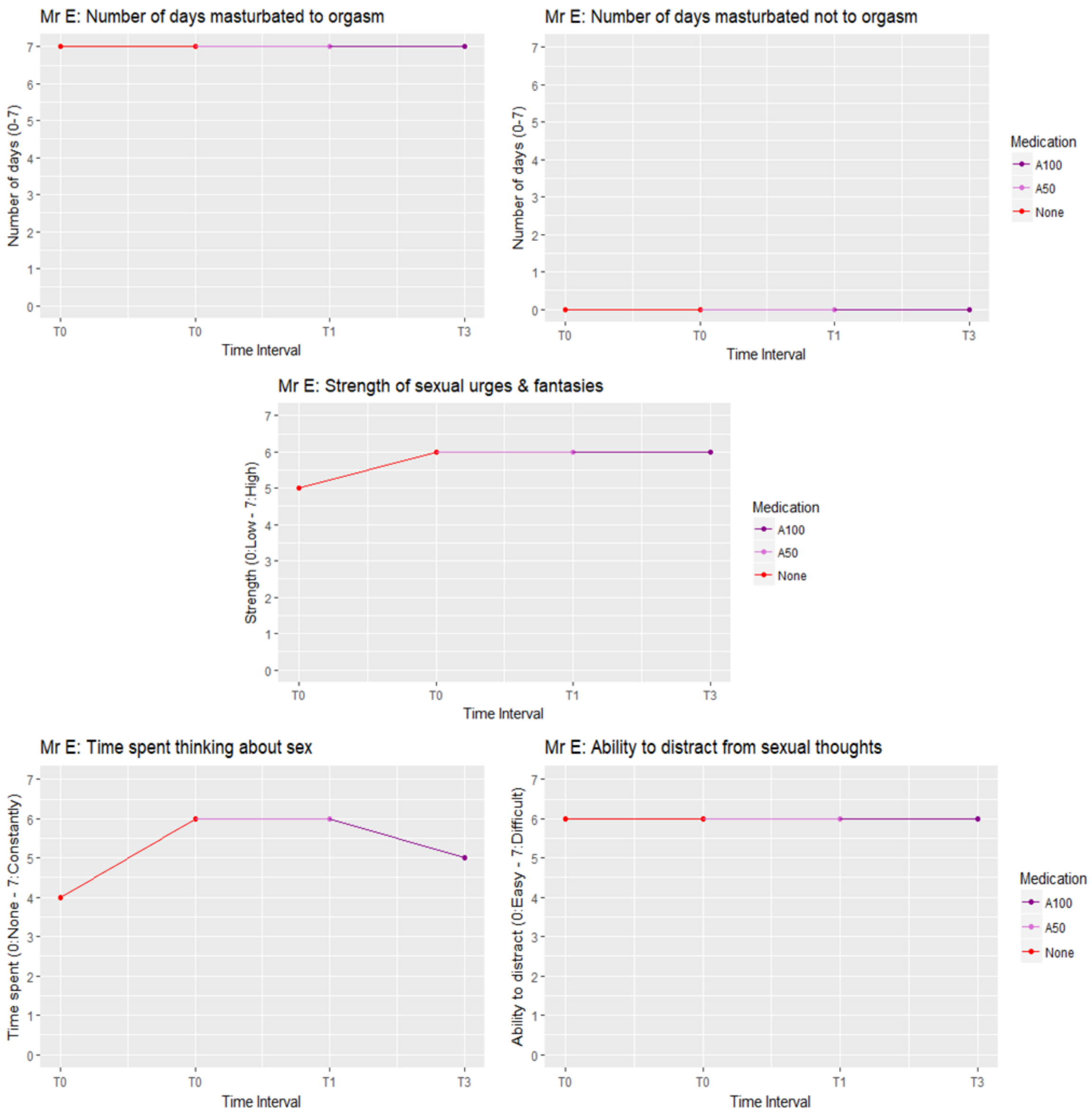

Figure 5. Changes in Mr E's clinical indicators of PSA

Mr E's case demonstrates an example of when medication alone is not sufficient to manage high levels of PSA. This may be in part due to the high levels of sexual behaviour and thoughts he was experiencing prior to taking any medication. Although at face value Mr E is reporting the same level of hypersexuality as others in the sample, the framing of the questions used in the clinical measures means that their comparability is limited. That is, for Mr E a score of seven may be indicative of a much higher level of sexual behaviour or thoughts than other participants who also are scoring seven, particularly based on the level of masturbation reported. This is because, for one participant, a score of seven could indicate seven total sexual outlets (i.e. masturbating once per day), whereas for Mr E this could equate to up to 105 outlets (given that he reports masturbating up to 15 times per day). In this sense, the clinical measures themselves may lack the necessary sensitivity to observe the nuance

in these behaviours, even among those exhibiting hypersexual behaviours. This is further discussed below.

Mr E's lack of progress on medication may also be due to his lack of psychological treatment as he has not developed any insight into his offending or awareness regarding the problematic levels of arousal with reports that he enjoys these. As such, it is a possibility that Mr E does not want these to reduce and may instead be taking the medication for alternative reasons, for example, to attempt to demonstrate a reduction in risk to the parole board, which was an extrinsic motivator discussed by individuals taking anti-androgens (Lievesley et al., 2024), and are typically associated with lower levels of engagement with treatment. It is also worth noting here that for Mr E, his treatment journey lasted only five months (just three of which were accompanied by clinical observations). This is potentially not long enough to witness significant changes on the types of measures used to examine clinical changes in PSA. At the very least there were no substantial changes in MMPSA dose or class which indicates the rapid discontinuation of treatment at the first opportunity, potentially alluding to a lack of motivation or readiness to change.

Pathway 6: Re-entry pathway

Case example: Mr F

History of PSA

Mr F reports frequent masturbation to images of both males and females from the age of nine, with his first sexual contact with another person aged 12 and regularly engaging in sexual contact with peers from the age of 13. While Mr F reports only two stable relationships. He is open about his past unfaithfulness and his casual attitude towards sex. In being so open, he reports having had over 100 sexual partners which is partly due to working as a prostitute for a short period.

Mr F reports having constant sexual thoughts and becoming easily aroused with a prolonged high frequency of masturbation - masturbating approximately 17 times per day while in the community five times per day while in custody. He reports this to be unmanageable and interfering in daily activities.

Treatment journey

Fluoxetine 20mg → None → Fluoxetine 20mg → Fluoxetine 40mg

Mr F was initially prescribed a daily dose of Fluoxetine 20mg due to high levels of PSA, particularly hypersexuality that demonstrated a compulsive element. This dosage resulted in reduced levels of both the psychological (i.e. sexual preoccupation) and behavioural (i.e. hypersexuality) facets of PSA. Despite some fluctuations in relation to his clinical measures of PSA, he remained on this dosage for approximately 12 months before stopping the medication as he felt he no longer needed it. After approximately one month of no medication, Mr F requested to go back onto the medication after reporting increases in relation to all clinical measures since stopping the medication (T10-T13). As such, he was restarted on Fluoxetine 20mg at T13. Mr F remained on this for approximately 19 months, demonstrating a reduction in all measures over the first 10 months (T13-T23). However, towards T32 Mr F noticed an increase in his levels of sexual thoughts and arousal, and his ability to manage these had decreased. This led to an increase in his levels of masturbation. As a result of this, his dosage was increased to Fluoxetine 40mg. This led to all clinical measures decreasing and stabilising at a low level from T32 onwards. Mr F was still on this dosage, and had been for approximately 20 months, at the point of data collection.

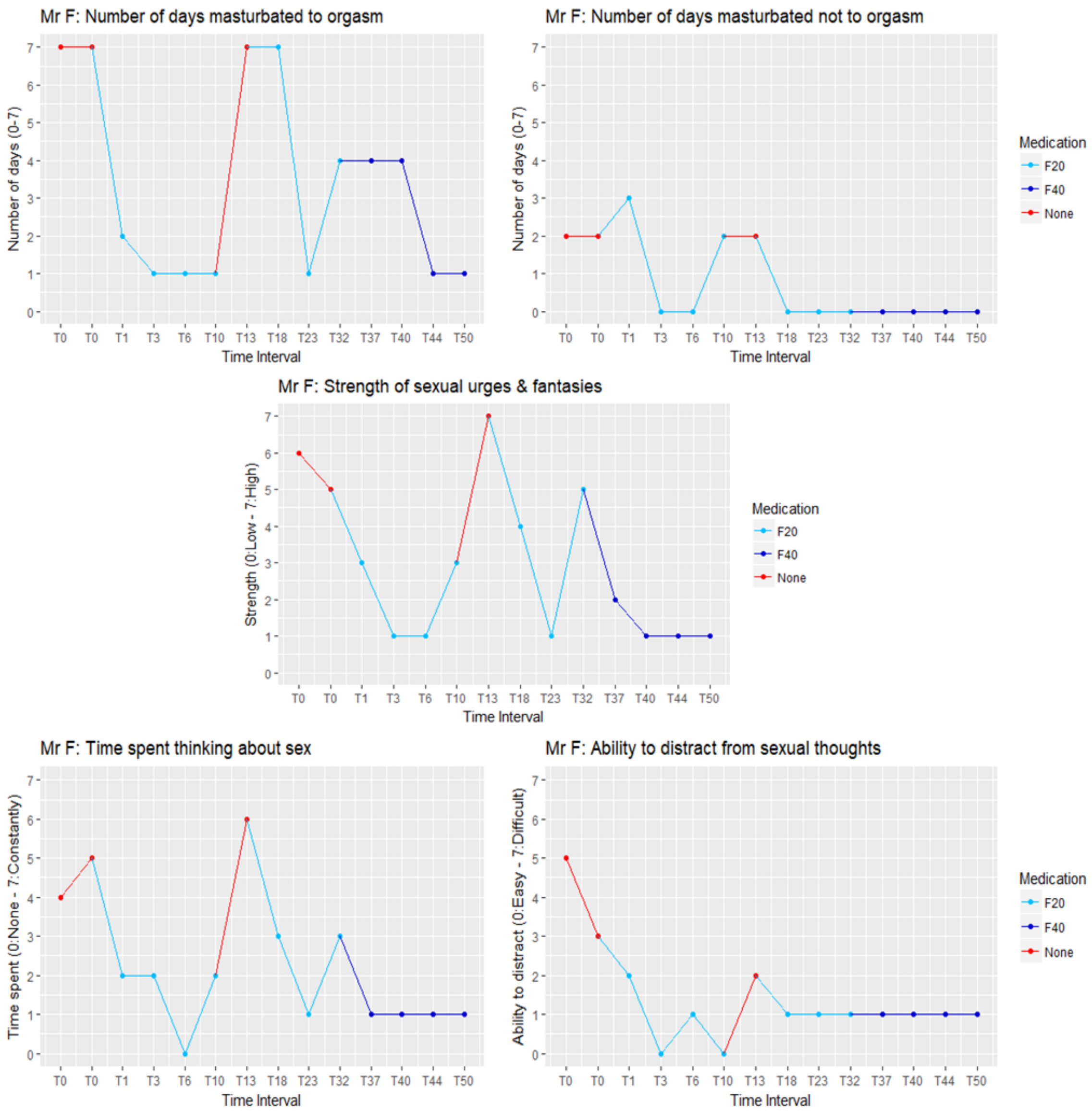

Figure 6. Changes in Mr F's clinical indicators of PSA

Mr F's case demonstrates a disrupted journey by stopping the medication prematurely with no intention to resume it. It is often the case in medicine that once the symptoms of a condition have reduced or ceased to be problematic, patients then discontinue treatment (Beebe et al., 2016; Manmohan et al., 2012). It is possible that the same effect is being observed in relation to Mr F. That is, he reported significant reductions across a range of clinical measures of PSA, with these being perceived to be at a low and manageable level for a prolonged period of time. Thus, he felt that this issue was no longer problematic, and that the medication was no longer needed. Once the medication was stopped and the symptoms returned (as observed through marked increases in the clinical measures of PSA) the need for medication was re-established. This may be a reflection of the lack of engagement in psychological treatment, meaning that Mr F has not developed the necessary skills and strategies to manage his PSA without the need for medication, further highlighting the need for

combined psychological and MMPSA treatment. This relapse was managed without any detrimental impact due to occurring within a prison setting which allowed Mr F to return to medication when needed. However, the possibility of this occurring in a less controlled environment (e.g. in the community following release from custody) could be a cause for concern. That is, in a less controlled environment there is undoubtedly an increased level of exposure to potentially sexual stimuli. These stimuli could then trigger an increase sexual thoughts and arousal. With this in mind, it may be advisable that service users such as Mr F receive careful monitoring, not only while they are taking MMPSA, but also after the discontinuation of the treatment. This is in line with recommendations made by Grubin (2018). Further, some degree of communication and joined-up service provision should be encouraged in order to facilitate the easy access of the medication in the community upon release if required.
